# Design of a Shock-Protected Structure for MEMS Gyroscopes over a Full Temperature Range

**DOI:** 10.3390/mi15020206

**Published:** 2024-01-30

**Authors:** Yingyu Xu, Jing Lin, Chunhua He, Heng Wu, Qinwen Huang, Guizhen Yan

**Affiliations:** 1 School of Computer, Guangdong University of Technology, Guangzhou 510006, China; 2 Science and Technology on Reliability Physics and Application Technology of Electronic Component Laboratory, China Electronic Product Reliability and Environmental Testing Research Institute, Guangzhou 510006, China; 3 Classroom Management Center, Guangdong University of Technology, Guangzhou 510006, China; 4 National Key Laboratory of Science and Technology on Micro/Nano Fabrication, Institute of Microelectronics, Peking University, Beijing 100871, China

**Keywords:** MEMS gyroscope, shock models, reliability design, thermal reliability

## Abstract

Impact is the most important factor affecting the reliability of Micro-Electro-Mechanical System (MEMS) gyroscopes, therefore corresponding reliability design is very essential. This paper proposes a shock-protected structure (SPS) capable of withstanding a full temperature range from −40 °C to 80 °C to enhance the shock resistance of MEMS gyroscopes. Firstly, the shock transfer functions of the gyroscope and the SPS are derived using Single Degree-of-Freedom and Two Degree-of-Freedom models. The U-folded beam stiffness and maximum positive stress are deduced to evaluate the shock resistance of the silicon beam. Subsequently, the frequency responses of acceleration of the gyroscope and the SPS are simulated and analyzed in Matlab utilizing the theoretical models. Simulation results demonstrate that when the first-order natural frequency of the SPS is approximately one-fourth of the gyroscope’s resonant frequency, the impact protection effect is best, and the SPS does not affect the original performance of the gyroscope. The acceleration peak of the MEMS gyroscope is reduced by approximately 23.5 dB when equipped with the SPS in comparison to its counterpart without the SPS. The anti-shock capability of the gyroscope with the SPS is enhanced by approximately 13 times over the full-temperature range. After the shock tests under the worst case, the gyroscope without the SPS experiences a beam fracture failure, while the performance of the gyroscope with the SPS remains normal, validating the effectiveness of the SPS in improving the shock reliability of MEMS gyroscopes.

## 1. Introduction

Given the extensive use of MEMS gyroscopes in smart devices, the automotive industry, aerospace, and various other sectors, these devices often confront challenging operational conditions such as high temperature, temperature cycling, and high-g shock [1,2,3]. Excessive shock loads can lead to reliability issues in MEMS gyroscopes, including structural fracture, particle contamination, short circuits, adhesion, and delamination [4,5,6,7]. Hence, it is crucial to develop a shock-protected structure to improve its applicability in high-g shock environments [8,9,10].

Common shock-resistant designs encompass optimized beam structures, buffer structures, energy dissipation mechanisms, and shock-protected packaging [11,12,13]. Peng et al. conducted shape optimization on MEMS cantilevers, comb structures, island beams, and folded beams, resulting in a 13–79% improvement in various shock resistance properties of MEMS inertial devices [14]. In terms of energy dissipation, researchers have proposed various shock absorber materials with a low coefficient of restitution (COR) as contact materials [15]. Eunhwan Jo et al. introduced a three-dimensional nano-composite material consisting of a vertically aligned carbon nanotube (CNT) array reinforced with ceramics for in-plane damping and energy dissipation in MEMS devices [16]. The nano-composite material demonstrated a significantly higher survival rate across a broad acceleration range (0–12,000 g) than control groups utilizing hard dampers and flexible spring dampers without CNT, with an increase of approximately 115%. Regarding shock-resistant packaging techniques, Yunbo Shi et al. developed an efficient encapsulation method employing three-layer wafer-level packaging for silicon chips [17]. This method effectively shields the transmission of stress waves, resulting in a measurement accuracy of better than 5% for sensors subjected to 200,000 g shock forces.

A buffer structure, designed for MEMS gyroscopes to withstand shocks, absorbs and disperses shock energy, thereby safeguarding sensitive structures and enhancing accuracy and sensitivity [18]. Currently, most theoretical investigations on MEMS shock-protected structures concentrate on Single-Degree-of-Freedom (SDOF) and Two-Degree-of-Freedom (TDOF) models. These models analyze the system’s response characteristics in the time domain by deriving dynamic equations [19,20,21]. Nevertheless, there is a significant lack of research on shock protection systems in the frequency domain. Moreover, temperature fluctuations induce thermal stresses leading to quality factor degradation, impacting the package reliability and shock resistance of MEMS devices [22,23,24]. Therefore, this paper proposes a shock protection structure to reduce the shock response peak in the full temperature range from −40 to 80 °C. The SDOF and TDOF models are established for the MEMS gyroscope with the shock-protected structure (SPS) to derive the shock transfer function. Moreover, the folded beam stiffness and the maximum positive stress are deduced. Finally, experimental tests are performed to verify the effectiveness of the theoretical and simulation analysis.

## 2. Modeling and Analysis

### 2.1. Single/Two Degree of Freedom Shock Models

**Figure 1:**
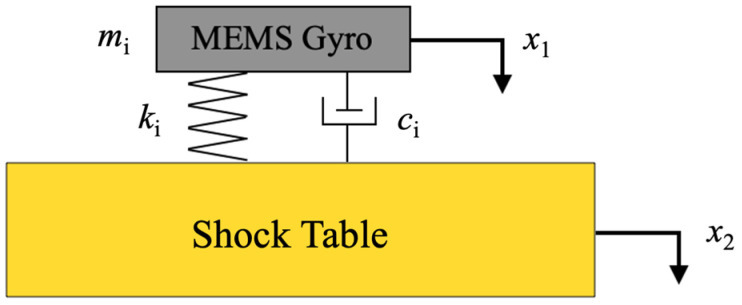
Schematic of the SDOF shock model.

**Figure 2:**
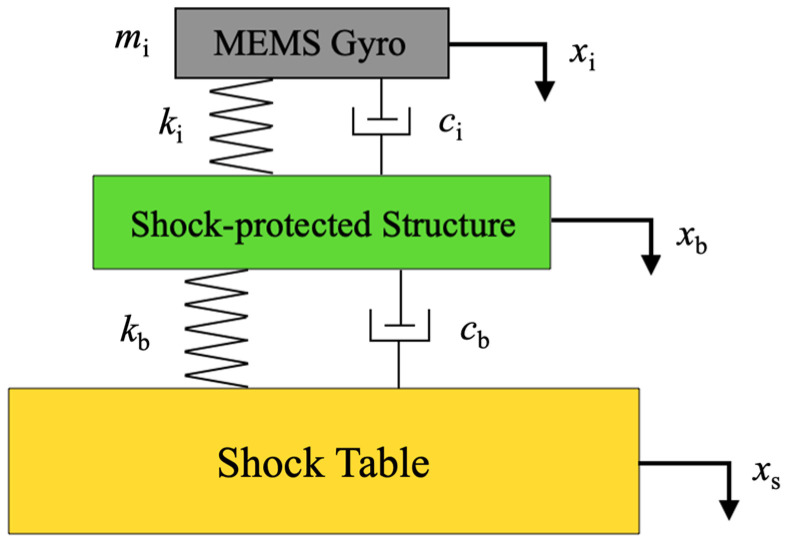
Schematic of the TDOF shock model.

Figure 1 illustrates the SDOF model of the MEMS gyroscope without the SPS. The gyroscope dynamics equation is as follows [25]:

(1) $$m_{i}{\ddot{x}}_{1}+c_{i}({\dot{x}}_{1}-{\dot{x}}_{2})+k_{i}(x_{1}-x_{2})=0$$

where *m*_i_, *c*_i_, and *k*_i_ represent the mass, damping force coefficient, and stiffness of the gyroscope, respectively. The shock stress is much larger than the electrostatic driving force, which is therefore ignored. *X*_1_ and *x*_2_ stand for the displacement of the gyroscope and the shock table, respectively. The transfer function is derived based on (1) using the Laplace Transform. The displacement ratio and acceleration ratio between the gyroscope structure and the shock table are calculated as follows:

(2) $$\frac{{\ddot{y}}_{1}}{{\ddot{x}}_{2}}=\frac{y_{1}}{x_{2}}=\frac{-m_{i}s^{2}}{m_{i}s^{2}+c_{i}s+k_{i}}$$

where *x*_2_ and ${\ddot{x}}_{2}$ are the displacement and acceleration *a*_0_ of the shock table, respectively. *Y*_1_ and ${\ddot{y}}_{1}$ are the relative displacement and relative acceleration *a*_r1_ of the MEMS gyroscope, respectively. Here, $y_{1}=x_{1}-x_{2}$, and ${\ddot{y}}_{1}={\ddot{x}}_{1}-{\ddot{x}}_{2}$. *S* is the complex variable. When the gyroscope is assembled with the SPS, it can be regarded as a TDOF shock model, as shown in Figure 2. Considering the effect of the SPS, the dynamics equations of the TDOF model are obtained as follows:

(3) $$(m_{i}s^{2}+c_{i}s+k_{i})y_{2}=-m_{i}s^{2}y_{3}-m_{i}s^{2}x_{s}$$

(4) $$(m_{b}s^{2}+c_{b}s+k_{b})y_{3}=-m_{b}s^{2}x_{s}+(c_{i}s+k_{i})y_{2}$$

where *m*_b_, *c*_b_, and *k*_b_ represent the mass, damping force coefficient, and stiffness of the SPS, respectively. The absolute displacement of the gyroscope, SPS, and shock table are marked with *x*_i_, *x*_b_, and *x*_s_, respectively. The relative displacement of the MEMS gyroscope is marked with *y*_2_, namely $y_{2}=x_{i}-x_{b}$. The relative displacement of the SPS is marked with *y*_3_, namely $y_{3}=x_{b}-x_{s}$. According to Equations (3) and (4), we can yield:

(5) $$\left(m_{b}s^{2}+c_{b}s+k_{b}\right)\left[\left(m_{i}s^{2}+c_{i}s+k_{i}\right)y_{2}+m_{i}s^{2}x_{s}\right]=m_{i}s^{2}\left[m_{b}s^{2}x_{s}-\left(c_{i}s+k_{i}\right)y_{2}\right]$$

The displacement ratio and acceleration ratio of the gyroscope structure to the shock table based on the TDOF model are determined as:

(6) $$\frac{{\ddot{y}}_{2}}{{\ddot{x}}_{s}}=\frac{y_{2}}{x_{s}}=\frac{-(c_{b}m_{i}s^{3}+k_{b}m_{i}s^{2})}{m_{i}m_{b}s^{4}+(c_{b}m_{i}+c_{i}m_{b}+c_{i}m_{i})s^{3}+(k_{b}m_{i}+k_{i}m_{b}+k_{i}m_{i}+c_{b}c_{i})s^{2}+(k_{b}c_{i}+k_{i}c_{b})s+k_{b}k_{i}}$$

where ${\ddot{y}}_{2}$ is the relative acceleration *a*_r2_ of the MEMS gyroscope and ${\ddot{x}}_{s}$ is the relative acceleration *a*_0_ of the shock table. Thus, Equation (5) could be transformed to:

(7) $$(m_{b}s^{2}+c_{b}s+k_{b})y_{3}+m_{b}s^{2}x_{s}=(c_{i}s+k_{i})y_{2}$$

By multiplying Equation (1) by (7), the displacement ratio and acceleration ratio of the SPS to the shock table can be obtained as:

(8) $$\frac{{\ddot{y}}_{3}}{{\ddot{x}}_{s}}=\frac{y_{3}}{x_{s}}=\frac{-\left[m_{i}m_{b}s^{4}+c_{i}(m_{b}+m_{i})s^{3}+k_{i}(m_{b}+m_{i})s^{2}\right]}{m_{i}m_{b}s^{4}+(c_{b}m_{i}+c_{i}m_{b}+c_{i}m_{i})s^{3}+(k_{b}m_{i}+k_{i}m_{b}+k_{i}m_{i}+c_{b}c_{i})s^{2}+(k_{b}c_{i}+k_{i}c_{b})s+k_{b}k_{i}}$$

where ${\ddot{y}}_{3}$ is the relative acceleration of the SPS. These theoretical models provide comprehensive insight into the displacement and acceleration response characteristics of the MEMS gyroscope with and without the SPS, contributing to the effective design and optimization of shock-protected structures for MEMS devices.

### 2.2. U-Shaped Folded Beam Model

**Figure 3:**
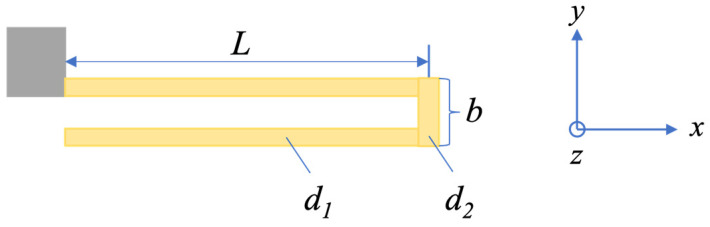
Structure of a U-shaped folded beam.

The mass block is supported by four elastic beams, which are the weakest links when subjected to external shocks. Therefore, it is necessary to analyze the stiffness and the maximum positive stress. The spring beams of the gyroscope are designed as U-shaped folded beams, as shown in Figure 3. The beam is characterized by length (*L*), thickness (*h*), width (*d*_1_), end beam length (*b*), and end beam width (*d*_2_), typically with $d_{1}=d_{2}$ and L<<b. The stiffness of a beam in the x-axis and z-axis is much greater than that in the y-axis, and usually the greater the stiffness, the stronger the impact resistance. Therefore, this paper focuses on the stiffness analysis of the weakest link, namely the stiffness analysis in the y-axis. The stiffness of the beam in the y-axis can be deduced according to the energy method as follows [26,27]:

(9) $$K=\frac{E_{0}hd_{1}^{3}(b+2L)}{4L^{3}(2b+L)}\approx \frac{E_{0}hd_{1}^{3}}{2L^{3}}$$

where *E*_0_ is the Young’s modulus of silicon. According to the above equations, *K* is inversely proportional to *L*^3^, proportional to *hd*_1_^3^, and independent of *b*. As the mass is supported by four elastic beams, $\omega_{i}=\sqrt{4K/m_{i}}$. The value of *ω*_i_ can be altered by adjusting parameters such as *L*, *h*, *d*_1_, or *m*_i_. Given that the shear stress in the beam is significantly lower than the tensile stress, this paper primarily examines shock resistance with a focus on positive stresses. The maximum positive stress (*σ*_max_) in the beam cross-section for motion in the y direction is [28]:

(10) $$\sigma_{\max}=\frac{M_{\max}u}{I}=\frac{F_{\max}Lu}{4I}$$

where *M*_max_ represents the maximum bending moment and *u* represents the distance from the surface to the neutral layer, namely, $u=d_{1}/2$. The moment of inertia of the beam section is $I=hd_{1}^{3}/12$. Considering the most extreme case, the maximum effective shock force $F_{\max}=m_{i}a_{\max}\approx 3m_{i}a_{0}/2$. *a*_max_ and *a*_0_ stand for the maximum response acceleration and external shock acceleration, respectively. According to Figure 10 below, the maximum shock response acceleration is approximately 1.5 times as big as the external shock acceleration. Thus, Equation (10) can be simplified as:

(11) $$\sigma_{\max}=\frac{3m_{i}a_{\max}L}{2hd_{1}^{2}}\approx \frac{9m_{i}a_{0}L}{4hd_{1}^{2}}$$

According to Equation (11), it can be seen that *σ*_max_ is proportional to *L* and inversely proportional to *hd*_1_^2^, which is opposite to the trend of stiffness. Increased stiffness or intrinsic frequency leads to reduced shock stresses in the beams. In addition, the highest stress will occur at the clamping. To solve this issue, rounded clamping can be adapted to distribute the stress more evenly and reduce the peak stress at the clamping point. The shock resistance of the beam can be evaluated by comparing the maximum stress with the yield strength of the silicon material.

## 3. Structural Design

To enhance the shock resistance of the MEMS gyroscope, a parameter optimization method can be adopted [29]. This includes modifying the structural parameters to significantly deviate the intrinsic frequency of the gyroscope from the shock frequency and reducing the quality factor and mass accordingly. Theoretically, increasing the stiffness or the natural frequency could lead to a reduction in the shock stresses in the beam, thus improving the shock resistance. However, these methods have the drawbacks of sacrificing mechanical sensitivity and signal-to-noise ratio [30]. In addition, the incorporation of flexible stopper structures contributes to a certain extent in enhancing shock resistance, but the inherent limitation of the small stopper structures is that silicon debris may be generated after collisions. This phenomenon significantly impacts the noise characteristics of the device. Aiming at solving these issues, an SPS is proposed in this section to minimize the influence of shock and improve reliability.

**Figure 4:**
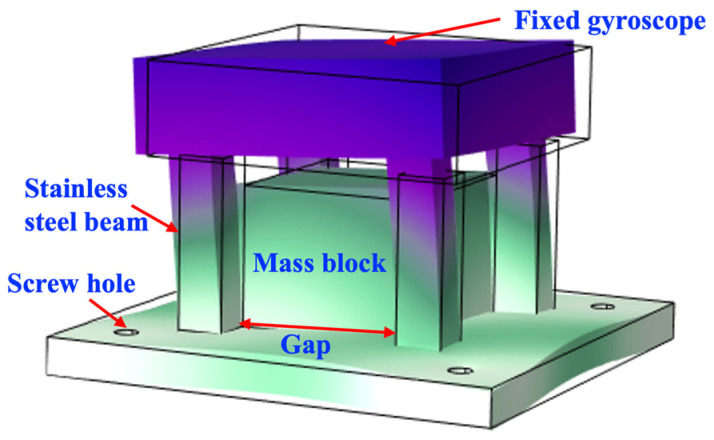
Structural schematic of the SPS for MEMS gyroscopes.

To effectively absorb and dissipate the shock energy, the SPS is designed to provide a reliable and stable platform for the MEMS gyroscope with a cube frame supported by four stainless-steel beams. The gyroscope is mounted on the top of the frame, and the SPS is secured to the substrate with four fixed holes, as depicted in Figure 4. A large mass block is integrated into the base of the frame to further enhance its shock-resistance capabilities. The resonant frequency of the SPS can be adjusted by changing the heights and gaps of the four beams or tuning the size of the mass block. Considering that the U-shaped folding beams may be horizontally or orthogonally distributed in the gyroscope structure, and the SPS is mainly designed to protect the motion in the x-y plane, the modes of the SPS in the x-y plane need to be carefully considered.

## 4. Simulation and Experiments

### 4.1. Simulation Analysis

The simulation analysis is performed using Matlab software R2022b. Based on Equations (2), (6), and (8), the frequency domain response of the displacement and acceleration of both the MEMS gyroscope and the SPS is analyzed separately. According to experience, the intrinsic frequency (*ω*_i_) of the MEMS gyroscope is set to 2π·8965.5 Hz, with a mass of *m*_i_ = 7 × 10^–7^ kg. The quality factors of the gyroscope and the SPS are marked with *Q*_i_ and *Q*_b_, which are set to 2000 and 100, respectively. Firstly, the effects of the SPS intrinsic frequency (*ω*_b_) on the shock characteristics are analyzed with constant *Q*_i_ and *Q*_b_. Furthermore, since the quality factor and resonance frequency are temperature-dependent, the frequency domain response characteristics of the shocks at different temperatures are analyzed by changing the parameter *ω*_i_.

**Figure 5:**
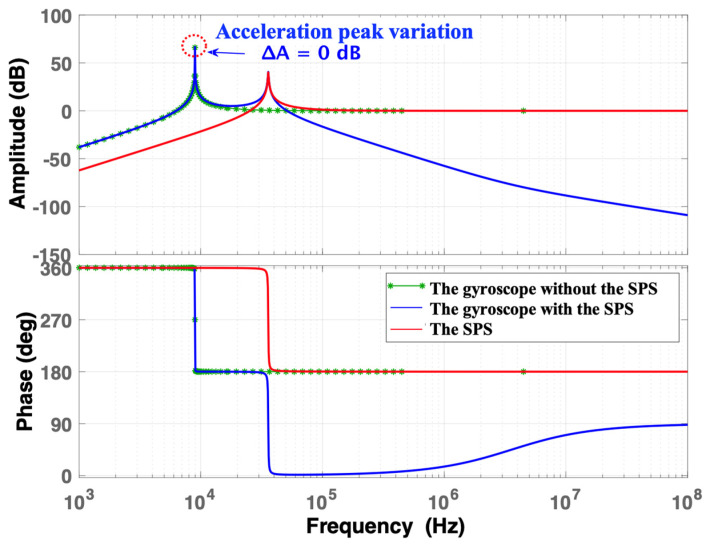
The frequency response of normalized acceleration when *ω*_b_ = *ω*_i_·4.

When *ω*_b_ = *ω*_i_·4, the frequency responses of the normalized acceleration (i.e., the ratio of the response acceleration to the shock acceleration) of the gyroscope, the gyroscope with the SPS, and the SPS are shown in Figure 5. ∆*A* is defined as the decrease in the acceleration peak of a gyroscope with the SPS compared with that of a gyroscope without the SPS. When the frequency is 8965.5 Hz, the acceleration response amplitude of the gyroscope with the SPS and that of the gyroscope without the SPS both show peaks that are similar in magnitude to each other. When the frequency is 35,862 Hz, the second peak of the acceleration response of the gyroscope with the SPS occurs due to the resonance of the SPS. The difference in the peaks of the SPS and the gyroscope with the SPS is not obvious. Subsequently, the amplitudes of the frequency responses of the gyroscope and SPS remain constant, while the amplitude of the gyroscope with the SPS gradually decreases with increasing frequency.

**Figure 6:**
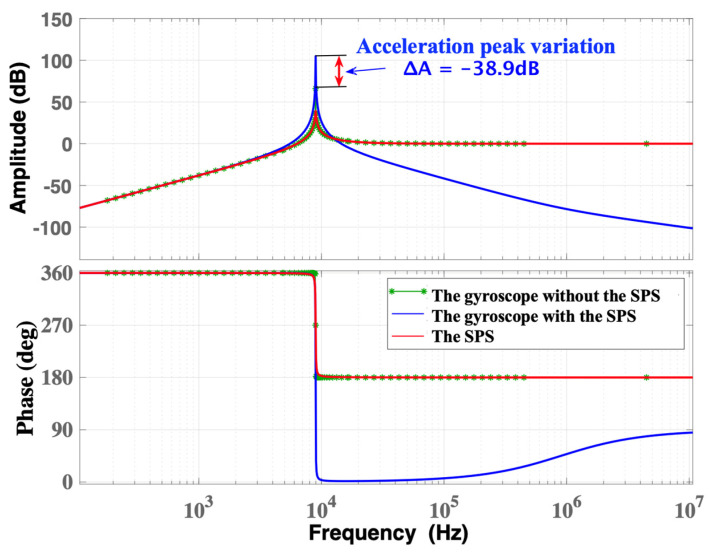
The frequency response of normalized acceleration when *ω*_b_ = *ω*_i_.

When *ω*_b_ = *ω*_i_, the frequency responses of the normalized acceleration are shown in Figure 6. At the frequency of 8965.5 Hz, the peak acceleration occurs simultaneously due to the resonances of the gyroscope and SPS, resulting in acceleration peaks of 66 dB and 39.2 dB, respectively. For the MEMS gyroscope with the SPS, the acceleration peak rises dramatically to 105 dB. This means that the shock acceleration response is amplified when the resonance frequency of the SPS is close to that of the MEMS gyroscope. Therefore, to enhance the shock resistance of the MEMS gyroscope, it is essential to prevent the natural frequency of the SPS from approaching that of the gyroscope. This strategy can effectively minimize the risk of resonance-induced amplification.

**Figure 7:**
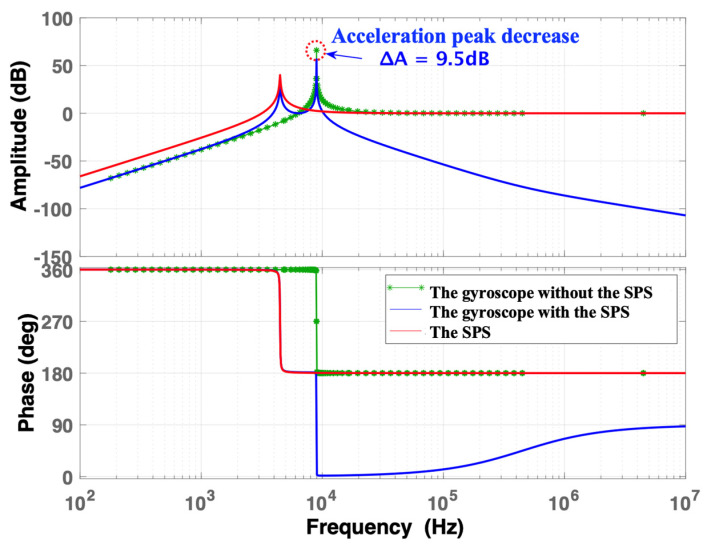
The frequency response of normalized acceleration when *ω*_b_ = *ω*_i_/2.

When *ω*_b_ = *ω*_i_/2, the frequency responses of the normalized acceleration are shown in Figure 7. When the frequency is 4482.75 Hz, the first acceleration peak of the gyroscope with the SPS decreases by 9.5 dB compared with that of the SPS. Similarly, the second acceleration peak of the gyroscope with the SPS decreased by 9.5 dB at 8965.5 Hz compared with that of the gyroscope. This indicates that reducing the natural frequency of the SPS to half of the gyroscope’s natural frequency can obviously decrease the acceleration peak. The resonant frequency of the SPS can be further reduced to achieve a lower acceleration peak.

**Figure 8:**
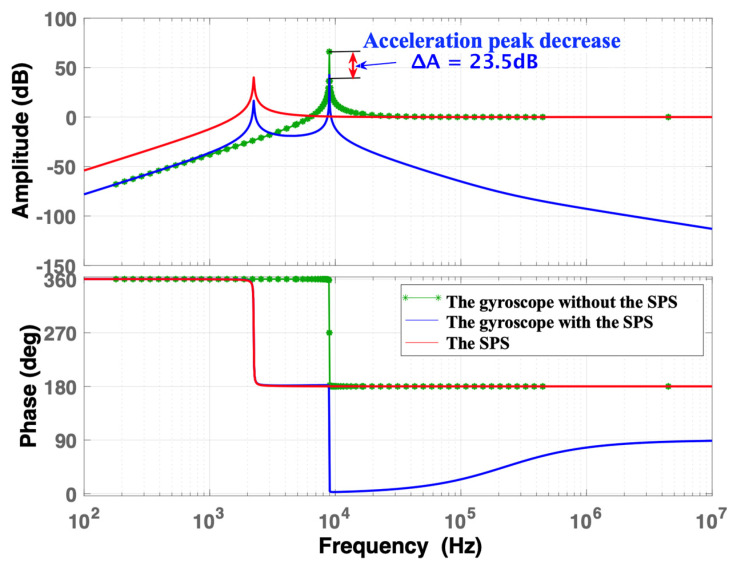
The frequency response of normalized acceleration when *ω*_b_ = *ω*_i_/4.

When *ω*_b_ = *ω*_i_/4, the frequency responses of the normalized acceleration are shown in Figure 8. The frequency response of the gyroscope with the SPS is similar to that of the gyroscope within the frequency range from 0 to 1000 Hz. It indicates that the application of the SPS has no effect on the low-frequency response of the gyroscope. In the mid-frequency region, a significant reduction of 23.5 dB is observed in the first peak of the frequency response of the gyroscope with the SPS, located at approximately 2241.4 Hz, when compared with that of the SPS. At 8965.5 Hz, the acceleration peaks of the gyroscope with the SPS and the gyroscope are 42.5 dB and 66 dB, respectively. However, the acceleration peak of the gyroscope with the SPS is effectively reduced by 23.5 dB, resulting in a decrease in amplitude of approximately 13 times. In the high-frequency region, the magnitude of the frequency response for the gyroscope with the SPS gradually decreases with increasing frequency, while those of the SPS and gyroscope maintain a relatively constant. It indicates that the SPS effectively absorbs high-frequency shocks, thereby providing substantial protection to the gyroscope.

**Figure 9:**
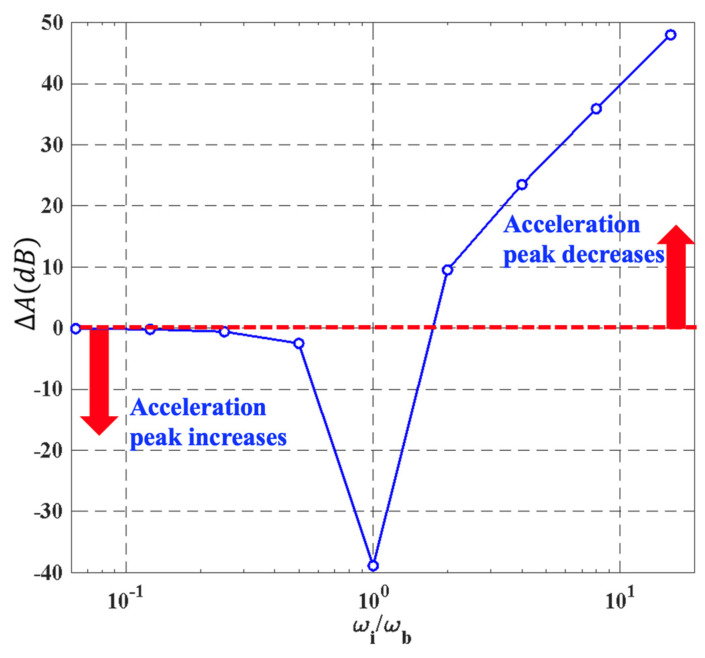
The acceleration peak varies with *ω*_i_/*ω*_b_. Above the red dotted line indicates that the acceleration peak decreases and the SPS serves as protection; Below the red dotted line indicates that the acceleration peak increases and the SPS does not serve as protection and is even harmful to the gyroscope.

Based on simulation analysis, it is evident that the SPS either amplifies or reduces the acceleration response of the MEMS gyroscope. From the above simulation cases, it is concluded that the MEMS gyroscope with the SPS has the lowest response peak when *ω*_b_ = *ω*_i_/4. In this case, the use of the SPS can efficiently alleviate the shock stress. To further study the relationship between the acceleration peak and *ω*_i_/*ω*_b_, a simulation analysis is conducted on a TDOF model. As shown in Figure 9, the acceleration peak varies with *ω*_i_/*ω*_b_, and the protected effect of the SPS gradually increases when *ω*_i_/*ω*_b_ is two or greater. However, the SPS has a counterproductive or no effect on the gyroscope when *ω*_i_/*ω*_b_ is less than 1. Specifically, if *ω*_i_/*ω*_b_ is equal to 1, the potential for shock-induced damage to the gyroscope reaches maximum. Therefore, to reduce the shock stress on the gyroscope, a sufficient decrease in the intrinsic frequency of the SPS is very significant. However, given that the frequency range of external vibration or impact acceleration is usually from 0 Hz to 2 kHz, in order to reduce the external disturbances and avoid resonance, the first-order mode of the SPS should be greater than 2 kHz. In addition, *ω*_i_ is 8965.5 Hz and *ω*_i_/4 is equal to 2241.4 Hz, which is larger than 2 kHz; hence, *ω*_i_/4 can be regarded as the optimized resonant frequency for the SPS.

**Figure 10:**
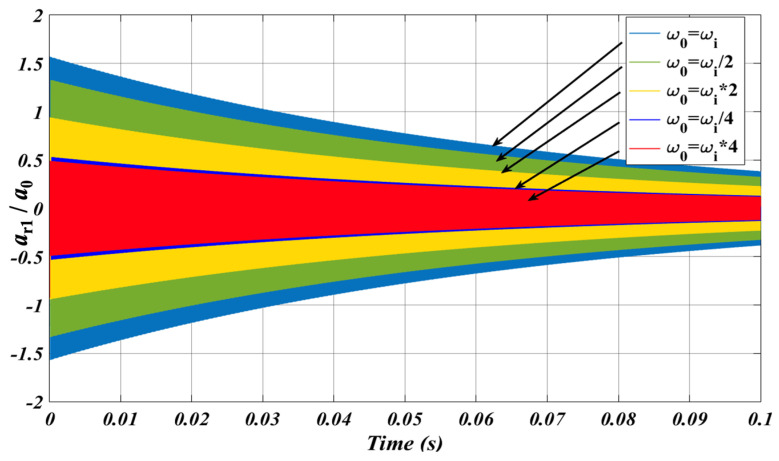
The curves of the normalized acceleration versus time under different *ω*_0_/*ω*_i_.

**Figure 11:**
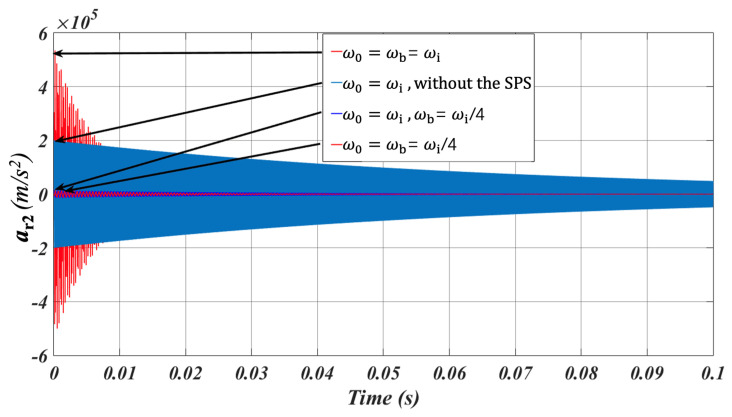
The curves of the response acceleration versus time under different cases.

In addition to the frequency-domain simulation, time-domain simulation based on the theoretical equations have also been conducted using Matlab Simulink (R2022b). For the simulation of the gyroscope without the SPS, the curves of the normalized acceleration versus time under different *ω*_0_/*ω*_i_ are shown in Figure 10. *ω*_0_ represents the shock frequency. It determines that the response acceleration is maximum when *ω*_0_ ≈ *ω*_i_, which is approximately 1.5 times as big as the shock acceleration. The response acceleration decreases as *ω*_0_/*ω*_i_ increases or decreases. For the simulation of the gyroscope with the SPS, considering the worst case, namely *ω*_0_ = *ω*_b_ = *ω*_i_, the gyroscope and the SPS both resonate. As shown in Figure 11, the maximum response acceleration reaches approximately 5.4 × 10^5^ m/s^2^. When *ω*_0_ = *ω*_i_ and without the SPS, the response acceleration of the gyroscope is as large as 2 × 10^5^ m/s^2^. It indicates that the response acceleration is obviously amplified when *ω*_b_ is approximate to *ω*_i_ and *ω*_0_. The response acceleration is minimized when *ω*_b_ = *ω*_i_/4. That is, the SPS effectively isolates the external shock and reduces the influence of the shock on the gyroscope.

**Figure 12:**
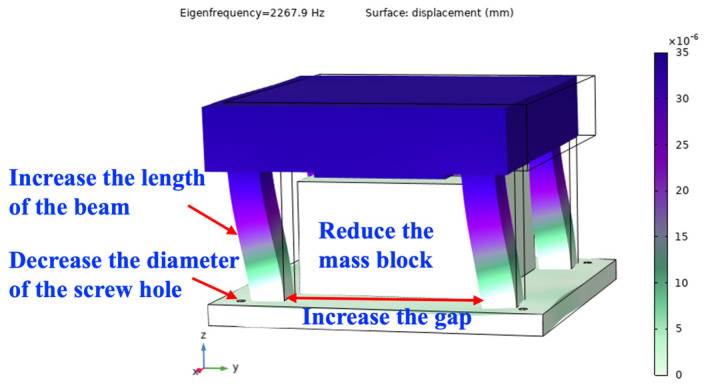
The first-order natural frequency of the SPS is finally adjusted to 2267.9 Hz.

Based on the analysis above, the first-order natural frequency of the SPS should be decreased to approximately *ω*_i_/4, namely 2241.4 Hz. To accomplish the goal, the lengths and the gaps of the four beams should be increased. Furthermore, the size of the mass block and the diameter of the screw hole should be decreased. By these means, the first-order intrinsic frequency of the SPS can be effectively reduced and finally adjusted to 2267.9 Hz, and it is simulated in Comsol software 6.1 and shown in Figure 12.

**Figure 13:**
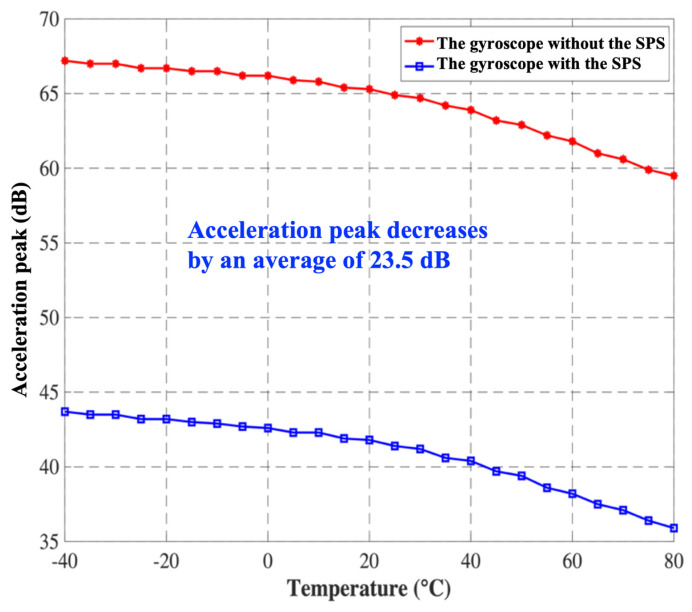
The acceleration peak varies with ambient temperature when *ω*_i_/*ω*_b_ = 4.

In order to analyze the shock-resistant characteristics of the SPS over the full temperature range, the shock simulations are conducted on the MEMS gyroscope with and without the SPS, and the simulation results are depicted in Figure 13. Since the natural frequency and quality factor of the gyroscope both decrease with the ambient temperature, the acceleration peaks of the gyroscope with and without the SPS both decrease with the ambient temperature. However, on average, the peak acceleration of the gyroscope with the SPS is reduced by 23.5 dB over the full temperature range, which is in accordance with the simulation result in Figure 8.

### 4.2. Experimental Verification

**Figure 14:**
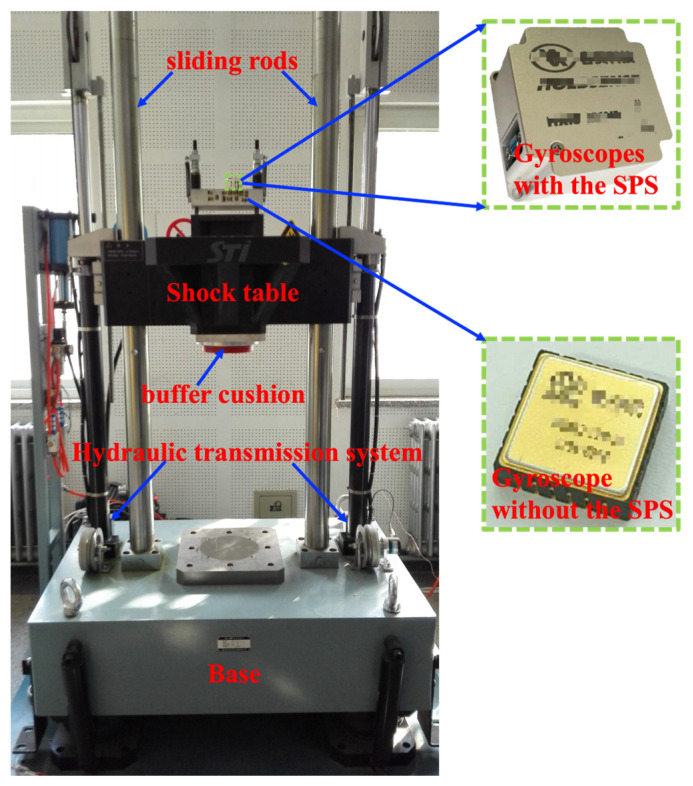
Shock experiments using the drop hammer impact test machine.

To evaluate whether the SPS affects the original performances of the gyroscope, the key performances of the gyroscopes with and without the SPS are measured. The MEMS gyroscopes are designed and fabricated by Peking University. The test results are listed in Table 1. It demonstrates that the bias instability and nonlinearity of the scale factor of the gyroscope with the SPS are a little better than those of the gyroscope without the SPS since there is a calibration and compensation design for the gyroscope with the SPS. Shock experiments on the MEMS gyroscope are carried out by a drop hammer impact test machine, as shown in Figure 14. The gyroscopes are mounted to the impact table. The drop hammer impact test machine mainly consists of a drop hammer, center console, and oil pump. The center console is used to control the drop hammer lifting and shock acceleration signals. The drop hammer has a large mass and is constrained on the sliding rods. The drop hammer is lifted and lowered by a hydraulic transmission system with a maximum height of 1.5 m. The material and thickness of the buffer cushion underneath the drop hammer can be used to adjust the impact pulse width. The oil pump is used to output hydraulic power. The drop hammer impact test machine can apply a maximum shock acceleration of 100,000 g with a pulse width of 5 μs to 2000 μs.

**Figure 15:**
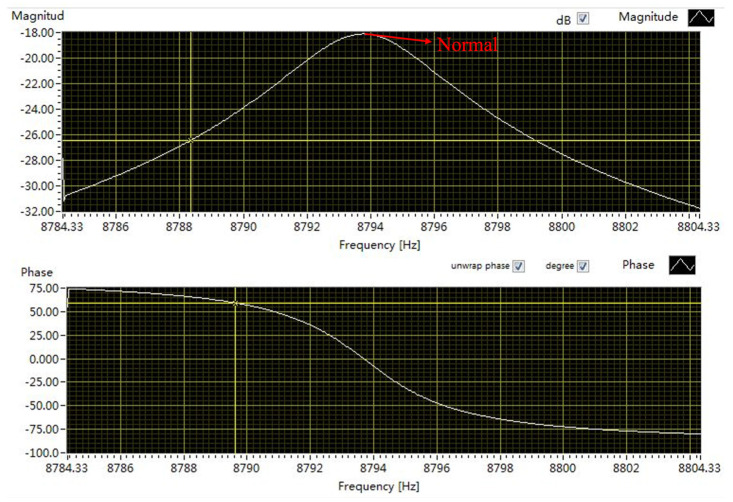
The frequency response curves of the gyroscope with the SPS after shock tests.

**Figure 16:**
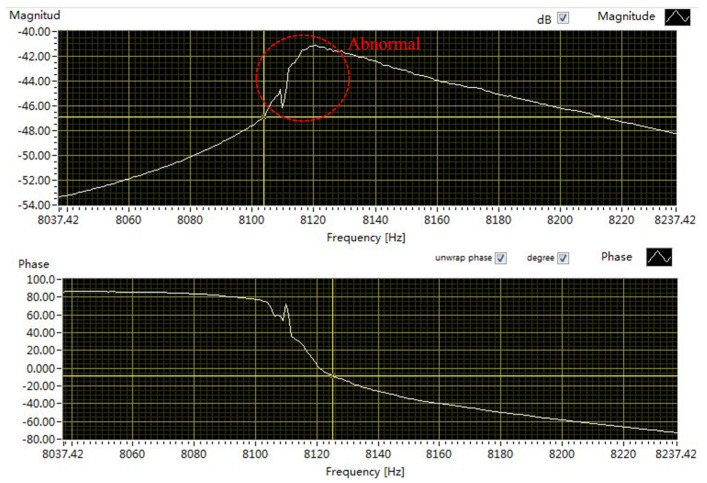
The frequency response curves of the gyroscope without the SPS after shock tests.

The quality factor of the vacuum-packaged gyroscope is 2000. To verify the shock resistance of the SPS, shock tests with an acceleration amplitude of 13,000 g and a duration of 56 μs are conducted on the gyroscopes with and without the SPS. The ratio of the *ω*_i_/*ω*_b_ for the gyroscope with the SPS is 4. Sweep-frequency tests of the gyroscope are required before and after the shock tests, and once the frequency response is abnormal, it is judged that the structure has failed. After the shock tests, the frequency response curves of the gyroscopes with and without the SPS are shown in Figure 15 and Figure 16, respectively. It is clear that the frequency response curves of the gyroscope with the SPS are normal while those of the gyroscope without the SPS are abnormal, indicating that the structure of the gyroscope without the SPS might be fractured.

**Figure 17:**
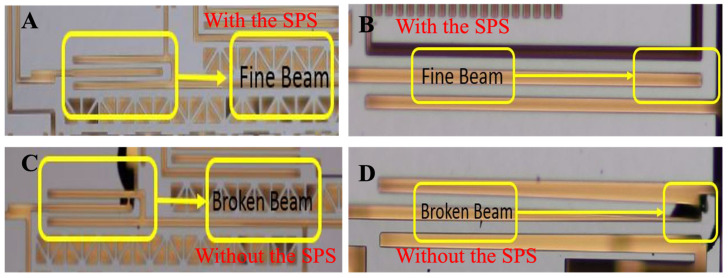
The metallographic micrographs of the microbeams after the shock tests: (**A**,**B**) with the SPS; (**C**,**D**) without the SPS.

Afterwards, a Destructive Physical Analysis (DPA) was applied to confirm the failure mode. After the encapsulation was opened, an internal visual inspection of the structure was conducted with a microscope. The metallographic micrographs of the microbeams after the shock tests are shown in Figure 17. The gyroscope without the SPS shows fracture failure of the microbeam. The ideal yield strength of silicon single crystals is approximately 7 GPa [3]. The maximum positive stress *σ*_max_ in the beam section can be calculated as 5.12 GPa according to Equation (10). For silicon beams, since the maximum positive stress is close to the yield strength, the larger cumulative damage under prolonged stress may lead to fracture failure. The end of the beam is a focal point for stress concentration, which may cause fractures. However, the beam of the gyroscope with the SPS remains intact, even though the shock frequency corresponding to the duration is close to the natural frequency of the gyroscope.

**Figure 18:**
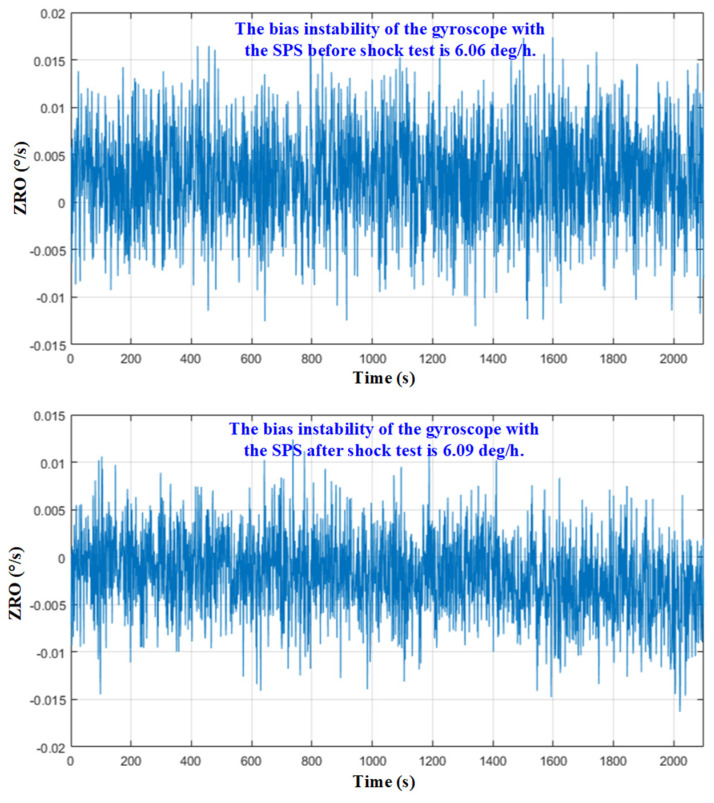
The ZRO of the gyroscope with the SPS before and after shock test.

Zero-rate output (ZRO) tests of the gyroscope with the SPS at room temperature were performed before and after shock tests, as shown in Figure 18. The bias instabilities of the gyroscope with the SPS before and after the shock test are 6.06 deg/h and 6.09 deg/h, respectively. They are very close, which further verifies that the gyroscope with the SPS after the shock test is intact and the SPS is effective.

## 5. Conclusions

In this paper, an SPS is proposed to enhance the shock resistance of MEMS gyroscopes over the full temperature range of −40 °C to 80 °C. The shock transfer functions of the gyroscope and the SPS are derived using Single Degree-of-Freedom and Two Degree-of-Freedom models, and the U-folded beam stiffness and maximum positive stress are deduced to evaluate the shock resistance of the silicon beam. Subsequently, the frequency responses of acceleration of the gyroscope and the SPS are simulated and analyzed in Matlab utilizing theoretical models. The simulation results demonstrate that when the first-order natural frequency of the SPS is approximately one-fourth of the gyroscope’s resonant frequency, the impact protection effect is best, and the SPS does not affect the original performance of the gyroscope. To reduce the first-order natural frequency of the SPS, the lengths and the gaps of the four beams should be increased, while the size of the mass block and the diameter of the screw hole should be decreased. The acceleration peak of the gyroscope is reduced by approximately 23.5 dB when equipped with the SPS in comparison to its counterpart without the SPS. The anti-shock capability of the gyroscope with the SPS is enhanced by approximately 13 times over the full temperature range. After the shock tests under the worst case (i.e., the shock frequency is equal to the gyroscope’s resonant frequency), the gyroscope without the SPS experiences a beam fracture failure, while the performance of the gyroscope with the SPS remains normal, validating the effectiveness of the SPS in improving the shock reliability of MEMS gyroscopes.

Due to the difficulty of establishing an experimental platform with both temperature and shock stresses, the current study only makes the simulation over the full temperature range without corresponding experimental verification. In the future, to further validate the effectiveness of the SPS, more shock experiments will be performed under different ambient temperatures. In addition, this work focuses on the analysis of the weakest link and only one axis. In the future, more simulation and experimental analyses will be conducted for the other two axes to comprehensively evaluate the impact protection capability of the SPS.

## Figures and Tables

**Table 1:** The key performances of the gyroscopes with and without the SPS.

Parameter	Without the SPS	With the SPS
Bias instability (°/h)	10	6
Bias instability at full temperature (°/h)	30	17
Nonlinearity of the scale factor (ppm)	100	15

## Data Availability

Data is contained within the article.
